# Supplementary material for: Timing Differences in Stride Cycle Phases in Retired Racehorses Ridden in Rising and Two-Point Seat Positions at Trot on Turf, Artificial and Tarmac Surfaces
Source: Animals (Basel). 2023 Aug 9;13(16):2563. doi: 10.3390/ani13162563 (PMC10451298; doi:10.3390/ani13162563)
Supplement: Supplementary file 1 [file animals-13-02563-s001.zip › animals-2483055-supplementary.pdf]

Article

Timing Differences in Stride Cycle Phases in Retired Racehorses Ridden in Rising and Two-Point Seat Positions at Trot on Turf, Artificial and Tarmac Surfaces

Kate Horan [1]*, Haydn Price [2], Peter Day [1], Russell Mackechnie-Guire [3] and Thilo Pfau [4]

Supplementary Information

Table S1 Vertical movement asymmetry data for horses trotting in-hand on tarmac summarized by the parameters MinDiff, MaxDiff, UpDiff and range of movement (ROM). L1 – lumbar vertebra 1, TC – left tuber coxae, RTC – right tuber coxae.

Variable	Horse	Stride time (ms)	Number of strides	MinDiff* (mm)	MaxDiff§ (mm)	UpDiff† (mm)	ROM‡ (mm)
Poll	1	656	22	-12.3	-8.6	3.7	60.6
Poll	2	714	9	-27.3	-2.7	24.6	79.3
Poll	3	658	20	-6.4	2.9	9.3	45.5
Poll	4	686	51	1.6	5.6	4.0	47.2
Poll	5	658	32	-28.0	-7.5	20.5	63.1
Poll	6	651	26	17.7	14.5	-3.2	62.3
Withers	1	656	22	1.3	-5.9	-7.3	55.8
Withers	2	714	9	-8.4	2.8	11.2	77.0
Withers	3	658	20	-9.8	0.4	10.2	58.0
Withers	4	686	51	-3.7	3.6	7.3	60.0
Withers	5	658	32	-15.1	-1.7	13.5	66.0
Withers	6	651	26	-4.5	5.7	10.2	54.1
L1	1	656	22	6.9	-1.5	-8.5	71.0
L1	2	714	9	-5.7	2.8	8.5	83.6
L1	3	658	20	-10.3	3.9	14.1	72.2
L1	4	686	51	0.1	2.2	2.2	69.7
L1	5	658	32	-5.1	5.2	10.4	73.9
L1	6	651	26	2.3	15.8	13.6	69.7
Sacrum	1	656	22	4.4	-2.0	-6.4	68.2
Sacrum	2	714	9	-7.7	-0.2	7.6	77.0
Sacrum	3	658	20	-6.9	4.7	11.6	62.1
Sacrum	4	686	51	-6.0	0.8	6.8	66.9
Sacrum	5	658	32	-6.5	6.6	13.1	70.0
Sacrum	6	651	26	7.0	18.3	11.3	72.4
LTC	1	656	22	-19.3	13.5	32.8	100.2
LTC	2	714	9	-37.4	8.4	45.9	115.4
LTC	3	658	20	-37.4	26.0	63.4	106.5
LTC	4	686	51	-23.6	22.6	46.2	105.6
LTC	5	658	32	-12.8	11.6	24.4	89.7
LTC	6	651	26	-8.0	26.1	34.1	87.0
RTC	1	656	22	35.4	-15.9	-51.3	104.9
RTC	2	714	9	22.8	-4.3	-27.1	101.5
RTC	3	658	20	17.9	-11.3	-29.2	85.0
RTC	4	686	51	19.9	-19.5	-39.4	98.4
RTC	5	658	32	6.8	0.9	-6.0	82.3
RTC	6	651	26	22.4	7.5	-14.9	87.9

*MinDiff is the difference between the two displacement minima reached at approximately mid-stance.

§MaxDiff is the difference between the two displacements maxima reached at approximately mid-aerial phase.
†UpDiff is the difference between the two upward movement amplitudes from mid-stance to mid-aerial phase.
‡ROM, or range of movement, is the maximum amount of vertical displacement, calculated as the difference between the most positive maximum and most negative minimum.

Table S2. Pairwise comparisons for jockey position +surface effects on landing duration.

Jockey position +Surface A	Jockey position +Surface B	Mean Difference (A-B)	Std. Error	df	Sig.	95% Confidence Interval for Difference (lower bound)	95% Confidence Interval for Difference (upper bound)
Rising+Grass	Rising+Tarmac	53.2	6.1	36.9	<0.001	33.9	72.4
	Rising+Artificial	-13.1	5.7	37.0	0.422	-31.1	4.9
	Two-point+Grass	-3.8	9.5	35.4	1	-33.8	26.2
	Two-point+Tarmac	48.8	6.0	33.7	<0.001	29.8	67.8
	Two-point+Artificial	1.4	6.3	35.5	1	-18.5	21.3
Rising+Tarmac	Rising+Grass	-53.2	6.1	36.9	<0.001	-72.4	-33.9
	Rising+Artificial	-66.2	4.9	32.8	<0.001	-81.6	-50.9
	Two-point+Grass	-57.0	9.9	38.3	<0.001	-88.0	-25.9
	Two-point+Tarmac	-4.3	5.4	34.1	1	-21.5	12.8
	Two-point+Artificial	-51.7	6.4	36.2	<0.001	-71.8	-31.7
Rising+Artificial	Rising+Grass	13.1	5.7	37.0	0.422	-4.9	31.1
	Rising+Tarmac	66.2	4.9	32.8	<0.001	50.9	81.6
	Two-point+Grass	9.3	9.4	38.4	1	-20.1	38.6
	Two-point+Tarmac	61.9	5.1	35.1	<0.001	45.8	78.0
	Two-point+Artificial	14.5	5.6	34.9	0.207	-3.1	32.1
Two-point+Grass	Rising+Grass	3.8	9.5	35.4	1	-26.2	33.8
	Rising+Tarmac	57.0	9.9	38.3	<0.001	25.9	88.0
	Rising+Artificial	-9.3	9.4	38.4	1	-38.6	20.1
	Two-point+Tarmac	52.6	9.5	37.1	<0.001	22.8	82.4
	Two-point+Artificial	5.2	9.1	36.8	1	-23.3	33.8
Two-point+Tarmac	Rising+Grass	-48.8	6.0	33.7	<0.001	-67.8	-29.8
	Rising+Tarmac	4.3	5.4	34.1	1	-12.8	21.5
	Rising+Artificial	-61.9	5.1	35.1	<0.001	-78.0	-45.8
	Two-point+Grass	-52.6	9.5	37.1	<0.001	-82.4	-22.8
	Two-point+Artificial	-47.4	6.1	37.0	<0.001	-66.5	-28.3
Two-point+Artificial	Rising+Grass	-1.4	6.3	35.5	1	-21.3	18.5
	Rising+Tarmac	51.7	6.4	36.2	<0.001	31.7	71.8
	Rising+Artificial	-14.5	5.6	34.9	0.207	-32.1	3.1
	Two-point+Grass	-5.2	9.1	36.8	1	-33.8	23.3
	Two-point+Tarmac	47.4	6.1	37.0	<0.001	28.3	66.5
